# A micromechanical comparison of human and porcine skin before and after preservation by freezing for medical device development

**DOI:** 10.1038/srep32074

**Published:** 2016-08-25

**Authors:** S. A. Ranamukhaarachchi, S. Lehnert, S. L. Ranamukhaarachchi, L. Sprenger, T. Schneider, I. Mansoor, K. Rai, U. O. Häfeli, B. Stoeber

**Affiliations:** 1 Department of Electrical and Computer Engineering, University of British Columbia, Vancouver, BC V6T 1Z4, Canada; 2 Faculty of Pharmaceutical Sciences, University of British Columbia, Vancouver, BC V6T 1Z3, Canada; 3 Institute for Food Technology and Bioprocess Engineering, Technische Universität Dresden, Dresden, 01062, Germany; 4 Faculty of Engineering, South Asian Institute of Technology and Medicine, Malabe, 10115, Sri Lanka; 5 Faculty of Mechanical Engineering, Technische Universität Dresden, 01062 Dresden, Germany; 6 False Creek Healthcare Center, Vancouver, BC V5Z 1C6, Canada; 7 Department of Mechanical Engineering, University of British Columbia, Vancouver, BC, V6T 1Z4 Canada

## Abstract

Collecting human skin samples for medical research, including developing microneedle-based medical devices, is challenging and time-consuming. Researchers rely on human skin substitutes and skin preservation techniques, such as freezing, to overcome the lack of skin availability. Porcine skin is considered the best substitute to human skin, but their mechanical resemblance has not been fully validated. We provide a direct mechanical comparison between human and porcine skin samples using a conventional mechano-analytical technique (microindentation) and a medical application (microneedle insertion), at 35% and 100% relative humidity. Human and porcine skin samples were tested immediately after surgical excision from subjects, and after one freeze-thaw cycle at −80 °C to assess the impact of freezing on their mechanical properties. The mechanical properties of fresh human and porcine skin (especially of the stratum corneum) were found to be different for bulk measurements using microindentation; and both types of skin were mechanically affected by freezing. Localized in-plane mechanical properties of skin during microneedle insertion appeared to be more comparable between human and porcine skin samples than their bulk out-of-plane mechanical properties. The results from this study serve as a reference for future mechanical tests conducted with frozen human skin and/or porcine skin as a human skin substitute.

The low availability or lack of freshly excised human skin specimens for biophysical and biomechanical research involving skin, including bite mark research^1^, wound healing^2^ and transcutaneous device testing of microneedles for example^3^, can be challenging for technological developments in many disciplines. Potential reasons for the difficulty in acquiring human skin for research and development work range from ethical considerations^1^ to a lack of donors. Mechanical properties of human skin can vary as a function of source (i.e., race, gender, age, and body location), state (i.e., fresh/live, frozen, and immersed in solution), and environmental conditions (i.e., temperature and relative humidity). The measured properties can also depend on the testing protocol which typically measures force versus distance relationships during tension, compression, and indentation. Much work over many decades has assessed the mechanical properties of human skin to identify the factors affecting the mechanical behavior of skin as a whole, as well as individual layers^4^,^5^,^6^,^7^,^8^,^9^,^10^. Mechanical properties have been tabulated previously^3^,^11^ to illustrate the large variability in the mechanical responses of skin.

Animal skin, especially from small mammals, has served as a common substitute for human skin^5^,^12^. However, different animal skin types possess significant anatomical and physiological differences compared to human skin^13^. The most accurate model for human skin was found to be porcine skin, from the perspectives of anatomy and physiology^1^,^2^, immunogenicity, cellular composition, and morphology^14^. This led to the assumption that porcine skin would also have similar mechanical properties to human skin. Many studies thus used porcine skin as a substitution for human skin^15^,^16^,^17^ without confirming the mechanical similarities of both skin types in controlled experiments. The first controlled mechanical comparison of human and porcine abdominal skin specimens was performed by Ranamukhaarachchi *et al*.^3^, where porcine and human skin samples were treated and evaluated using identical test protocols. It was found that mechanical differences exist between porcine and human skin. For example, the porcine stratum corneum (SC) showed significantly lower Young’s moduli (both in-plane and out-of-plane) compared to human SC at physiological humidity conditions. The mechanical properties of human and porcine skin also changed differently when humidity increased from dry to wet conditions, which might have occurred due to structural differences between the two skin types. Though similar in anatomy and composition, subtle differences in the porcine skin structure may contribute to mechanical differences, as listed in Table S1 (Supplement Material).

For storage, skin is typically frozen to preserve the skin’s mechanical properties without inducing biological decomposition and structural changes^11^. Compared to other skin preservation methods, such as using formaldehyde for histology or embalming dead bodies, freezing induces the least structural and mechanical changes to skin. Micozzi (1986) showed that freezing caused mechanical disruption of skin and connective tissues leading to a decrease in stiffness in rat skin^18^. Foutz *et al.*^12^ showed that freezing at −70 °C did not affect the in-plane Young’s modulus, loading response and the ultimate tensile strength of rat skin, but significantly lowered its fracture strength^12^. More recently, in a non-mechanical characterization of skin, Mansoor *et al.*^19^ showed that freezing increased the diffusivity of drugs in porcine skin, most likely due to ice crystal formation during the freezing process, which subsequently led to structural damage, increased porosity, and potentiality to changes in the mechanical properties of the skin. The diffusion coefficient of doxorubicin was higher in frozen and thawed porcine skin compared to freshly excised porcine skin. The observations by Mansoor *et al.*^19^ agreed with the findings of Kasting and Bowman (1990), who found that the permeability of sodium ions in fresh human skin was significantly lower than in previously frozen human skin^19^,^20^. Since most indications on the likelihood of mechanical changes in human skin due to freezing were derived from small animal and non-mechanical studies, there is a need to determine the effect of freezing on the mechanical properties of human skin directly.

The mechanical properties of skin have a great impact on how microneedles can be applied to the skin surface. Microneedles are sub-millimeter needle projections that are used in transdermal medical applications, such as drug delivery, liquid extraction, and therapeutic drug monitoring. Microneedles function by disrupting the outer-most barrier layer of human skin (the SC) to access the viable epidermal and dermal skin layers. Microneedles are insertion-tested on skin as a quality control measure and to demonstrate successful penetration through the SC. Only a small number of microneedle insertion tests were conducted over the past decade directly in alive humans^21^,^22^ or on freshly excised human skin^3^ due to challenges in accessing these human skin tissues. The majority of other skin indentation tests have been conducted using human cadaver skin^3^,^23^,^24^, frozen and thawed human skin^25^, fresh porcine skin^19^, frozen and thawed porcine skin^26^,^27^, and other animal skins^28^,^29^. As a result, the microneedle insertion characteristics from these studies are challenging to compare with each other and are not able to fill the gaps in the knowledge surrounding the interactions between skin and microneedles.

The objectives of the present study were to provide a direct mechanical comparison between human and porcine skin to test the assumptions of mechanical similarity between the two skin types; and to assess the impact of freezing the skin on its mechanical properties. This mechanical comparison between human and porcine skin will serve as a reference for mechanical studies involving the two skin types, and assist in identifying the conditions where human skin can be simulated using porcine skin.

## Results and Discussion

**Figure 1:**
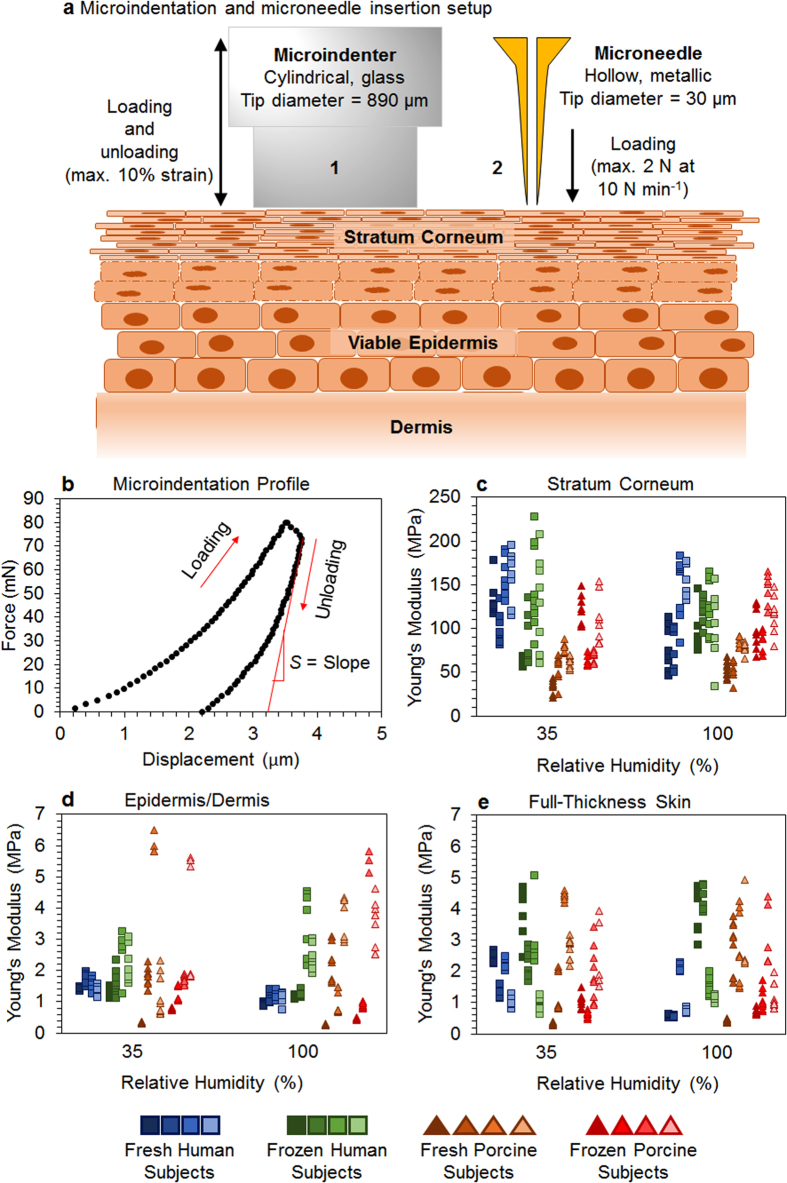
Microindentation of skin layers. A cylindrical microindentation tip (**a**-1; 0.89 mm diameter) loaded and unloaded a force into/from the skin surface. A hollow microneedle (**a**-2; 30 μm tip diameter) was inserted into skin at 10 N min^−1^ to a load of 2 N. The initial slope of the microindenter unloading (**b**), for example, from fresh human stratum corneum at 100% RH, was used to estimate the stiffness *S* of the skin layer to determine the Young’s modulus. The out-of-plane Young’s moduli of stratum corneum (**c**), epidermal/dermal composite (**d**) and full-thickness skin (**e**) were determined for human and porcine skin before and after freezing at −80 °C for 48 hours (four human, four porcine subjects were tested at 35% and 100% RH; n = 8 per subject).

Microindentation and microneedle insertion profiling were used to assess and compare the mechanical properties of skin (see Fig. 1a). Skin samples were distinguished by source (human vs. pig), state (freshly excised vs. frozen and thawed), and the relative humidity (RH) condition used during testing and analysis. Microindentation and microneedle insertion provided completely different mechanical characteristics of the skin, and provided greater insight into the anisotropic and heterogeneous nature of skin. During microindentation, the skin layers (individually or composite) are compressed by the microindenter; and the compressive strength (out-of-plane) of the skin layer dominantly impacts the resulting Young’s modulus measurement. In contrast, during microneedle insertion, tensile strength (in-plane) of the skin layers impart a dominant effect on the resulting mechanical properties, as described later.

The Young’s moduli of the stratum corneum (*E*_SC_), of the viable epidermis/dermis (*E*_ED_), and of the full-thickness skin (*E*_FT_) are shown in Fig. 1 for skin specimens from all human and porcine subjects in this study. Due to inherent and significant subject-to-subject variability among human and porcine subjects, analysis of variance with a three-factor factorial experiment in complete randomized arrangement of treatments (ANOVA; Table S2 provided in the Supplement Material) and Fischer’s Protected Least Significant Difference analysis (LSD; Table 1) were conducted by pooling data from all four subjects per skin source (i.e., human vs. pigs) into individual data sets.

### Young’s modulus of the stratum cornea

The mean *E*_SC_, extracted from microindentation profiles (Fig. 1b), ranged between 108–139 MPa for human skin and 56–111 MPa for porcine skin, which is comparable to other mechanical studies of skin where the *E*_SC_ ranged from 5–1,000 MPa^17^,^30^. In general, the *E*_SC_ was found to be significantly higher in human skin compared to porcine skin in our study (Fig. 1c, Table 1). The *E*_SC_ of fresh human skin decreased from 139 MPa (35% RH) to 111 MPa (100% RH), but the *E*_SC_ of fresh porcine skin increased from 56 MPa (35% RH) to 67 MPa (100% RH). This opposite effect of decrease/increase in *E*_SC_ of human and porcine skin for increasing RH indicated potential structural, compositional and/or functional differences between human and porcine skin that influence moisture retention of the skin (especially at low RH) and its consequential mechanical properties (see Table S2, Supplement Material; *P* = 0.007), as previously suggested by Ranamukhaarachchi *et al.*^3^. According to Silva *et al.*^16^, moisture is predominantly retained by the corneocytes of the SC at low RH, yielding a higher *E*_SC_; whereas, both corneocytes and SC lipids swell substantially at high RH yielding a lower *E*_SC_ due to softening of the SC. This behavior was observed in human SC, but not in porcine SC (Table 1)^16^.

Freezing affected human and porcine SC in different ways, highlighting potential structural and compositional differences between the two skin sources. A strong statistical significance was identified for the interaction between skin source and state (Table S2, Supplement Material; *P* < 0.0001), as freezing decreased the mean *E*_SC_ for human skin from 124 MPa to 109 MPa; but increased the mean *E*_SC_ for porcine skin from 62 MPa to 101 MPa. After freezing, human and porcine skin yielded a similar mean *E*_SC_ at high RH (~111 MPa; Fig. 1c), and that was also similar to the *E*_SC_ of fresh human skin, showing that porcine SC became a closer model to the human SC after freezing. The *E*_SC_ of most human skin samples decreased at 35% RH after freezing, but not at 100% RH. A possible explanation for the decrease in the *E*_SC_ of human skin due to freezing is structural damage caused by ice crystal formation to the SC, leading to disruption of the cell membranes and weakening of the intra-cellular bonds in the SC. The *E*_SC_ of porcine skin increased at both RH conditions after freezing, yielding a weak statistically significant relationship between the state of skin and RH (*P* = 0.0194). The RH conditions did not influence the *E*_SC_ of human skin post-freezing, possibly due to the impact of the freeze-thaw cycle on the nature of and components involved in moisture handling by the SC. In contrast, the *E*_SC_ of porcine skin increased with freezing under both RH conditions, likely due to differences in freeze-damage compared to human skin. A possible impact of ice crystal formation may be the rupturing of porcine corneocyte cell membranes during freezing, which hinders the water-retaining ability and reduces water-soluble hygroscopic materials in cells. According to Park and Baddiel (1972), such a destruction of the cell membrane resulting in loss of hygroscopic material can lead to the collapse of protein networks in the SC, providing a more compact structure with a higher elastic modulus^31^. More information on the statistically significant interactions between the different treatment methods tested in our study can be found in the Supplement Material.

### Young’s modulus of skin composites

Microindentation of epidermal/dermal (ED) composites (SC layer removed) showed a significant difference in the *E*_ED_ between human and porcine skin (Table 1; *P* = 0.024). The RH did not affect the *E*_ED_ significantly when all human and porcine samples were considered (Table 1). However, increasing RH did appear to decrease the *E*_ED_ of only human skin from 1.46 MPa at 35% RH to 1.06 MPa at 100% RH (*P* < 0.0001). Freezing increased the *E*_ED_ of human skin at 35% RH (*P* < 0.001) and at 100% RH (*P* < 0.0001), and of porcine skin samples at 100% RH (*P* = 0.02); but not at 35% RH (*P* = 0.95).

The *E*_FT,_ which was similar in magnitude to previously published results^7^,^32^,^33^, was not significantly influenced by skin type, state, or RH. A strong statistical significance was observed for the interaction between skin source and state on the *E*_FT_ (*P* < 0.0001). The *E*_FT_ of human skin increased from 1.31 MPa to 2.46 MPa after freezing; but decreased in porcine skin from 2.11 MPa to 1.37 MPa after freezing (Fig. 1e). Although RH did not impact the *E*_FT_ across all skin samples, a significant decrease in the *E*_FT_ from 1.69 MPa (35% RH) to 0.93 MPa (100% RH) was observed in fresh human skin (*P* < 0.0001), which followed a similar trend as fresh human SC (Fig. 1c). The *E*_FT_ of human skin increased due to freezing, while the opposite was observed for the *E*_FT_ of porcine skin, indicating differences in the structural changes undergone in human and porcine skin during freezing.

The *E*_ED_ (Fig. 1d) for fresh human skin followed the same trend as *E*_SC_ (Fig. 1c) and *E*_FT_ (Fig. 1e) as a function of RH, but the magnitude of change for *E*_ED_ was smaller. Therefore, the SC appeared to strongly affect the mechanical behavior of full-thickness skin. Similarly, in fresh porcine skin, the *E*_SC_ appeared to have a more prominent influence (compared to *E*_ED_) on the *E*_FT_, as *E*_SC_ and *E*_FT_ followed a similar trend as a function of RH. The *E*_ED_ of frozen human skin was lower than its *E*_FT_ by ~21% at both RH conditions (Fig. 1e), directly showing the impact of the SC on the *E*_FT_. Comparable trends for *E*_SC,_ *E*_ED_, *E*_FT_ of frozen human skin as a function of RH showed that freezing affected the skin layers in the same manner, and resulted in samples with out-of-plane mechanical properties that were unaffected by RH in their elastic range. In contrast, the *E*_ED_ of frozen porcine skin was higher than its *E*_FT_ by ~32% at 35% RH, and ~103% at 100% RH. Unlike for fresh porcine skin, the impact of the SC on the mechanical behavior of full-thickness frozen porcine skin remained unclear.

### Microneedle insertion profiling

**Figure 2:**
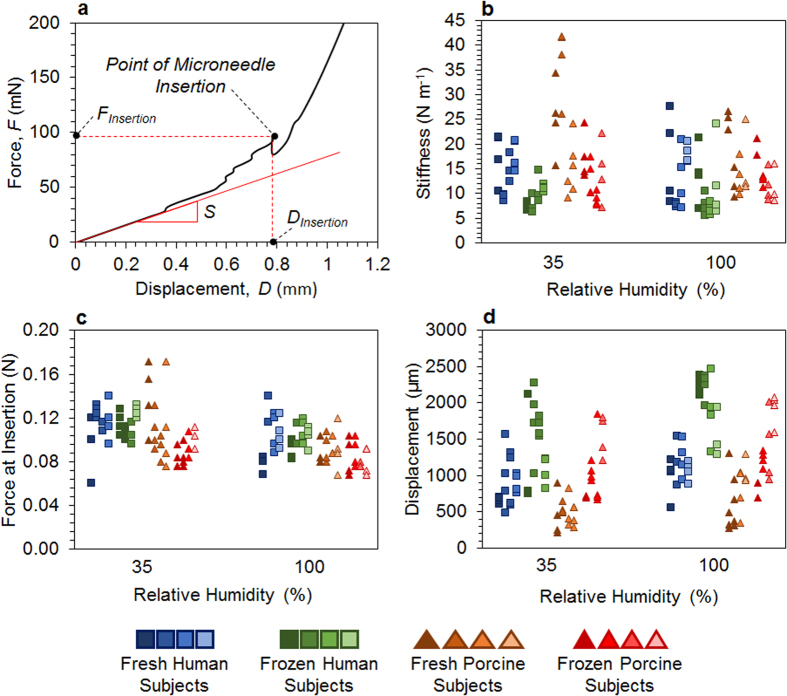
Microneedle insertion profiling in skin layers. Force versus displacement data from a typical microneedle insertion profile (**a**) was evaluated for stiffness (**b**), force of insertion (**c**), and displacement at insertion (**d**) parameters for human and porcine skin before and after freezing at −80 °C for 48 hours (four human and four porcine subjects were tested at 35% and 100% RH; n = 8 per subject).

Microneedle insertions into human and porcine skin provided key metrics to assess the in-plane mechanical behavior of skin (Fig. 2a), including 1) stiffness (*S*) during initial contact between the skin and the microneedle; 2) force required to break the SC by a microneedle (*F*_Insertion_), which correlated with the ultimate tensile strength (UTS) of the SC^3^; and 3) displacement (*D*_Insertion_) of a microneedle from the skin surface until SC rupture (including the skin deflection during stress application). The product of force and displacement at insertion yielded the work performed by a microneedle on skin to break the SC. The *S*, *F*_Insertion_, and *D*_Insertion_ results obtained from microneedle insertions were plotted in Fig. 2 for all skin specimens employed in this study. Due to significant subject-to-subject variability among human and porcine subjects, a three-factor factorial ANOVA (Table S4, Supplement Material) and a LSD analysis (Table 2) were conducted by pooling data from all four subjects per skin type into individual data sets.

Major differences between microindentation and microneedle insertion lie in the scale of the indentation devices and orientation of deformation yielding mechanical responses. Both microneedle and microindentation tip impart a compressive stress onto the skin sample in a region similar to their contact area. As the skin is being compressed, an in-plane tensile stress develops in the skin around the circumference of the indentation devices, in particular in the SC. Due to their very different tip diameters of 30 μm for the microneedle and 890 μm for the microindentation probe, the surface area-to-circumference ratios of the microindentation probe is 30 times larger than that of the microneedle (Fig. 1a). Hence, the mechanical response measured by the microneedle corresponds more to the tensile stress in the SC around its circumference, while the response measured by the microindentation probe is mainly caused by skin compression achieved with its relatively larger contact surface. It is suspected that during microneedle insertion, the impact of corneodesmosomes, a class of proteins responsible for ensuring cell-to-cell adhesion in the skin, on the mechanical strength of skin were more likely captured by the microneedle tip than during microindentation^34^. A microneedle tip likely deformed the skin directly on or very close to tight junctions that are formed by corneodesmosomes between corneocytes in the SC to contribute immensely to its mechanical and extreme-barrier properties^35^. They undergo degradation due to proteolytic activity of enzymes and inhibitors, affecting the localized mechanical properties of skin that impact microneedle insertions. Their degradation is dependent on surrounding humidity and moisture content in skin, and can be facilitated by freezing^36^.

The mean *S* of human skin tested during microneedle insertions was significantly higher (16.96 N m^−1^) than that of porcine skin (12.33 N m^−1^; Table 2; *P* < 0.0001), which was similar to the *E*_SC_ results (Fig. 1c). In fresh porcine skin, *S* decreased by 36% (from 24.4 N m^−1^ at 35% RH to 16.1 N m^−1^ at 100% RH; *P* < 0.01), due to swelling of corneocytes and SC lipids during hydration; such a significant decrease was not observed in fresh human skin. The *S* of fresh human and porcine skin was significantly different at lower RH (9.1 N m^−1^ difference; *P* = 0.0044), but not at higher RH (1.7 N m^−1^ difference; P = 0.45), showing that high RH facilitated similar localized behavior in both fresh human and porcine skin. RH did not impact *S* in frozen skins. Freezing decreased the *S* of human and porcine skins from 17.55 N m^−1^ (fresh) to 11.74 N m^−1^ (frozen), clearly indicating freezing-induced disruption of the SC structure. In contrast, freezing increased the *E*_SC_ during microindentation (Table 1), which suggested that there are major differences between in-plane and local mechanical responses versus out-of-plane and bulk mechanical responses of the skin due to freeze-damage. The decrease in the localized stiffness of skin after freezing is attributed to corneodesmosomal degradation, which compromised the integrity of cellular junctions in the SC.

The mean *F*_Insertion_ obtained in this study ranged between 0.104–0.111 N for human skin and 0.083–0.118 N for porcine skin, and was similar in magnitude (0.1–3 N) to a number of previous studies^22^,^25^,^29^. This *F*_Insertion_ was significantly dependent on all sample attributes explored in this study – skin type, state and RH (Table S4; *P* < 0.0001). A significant interaction between the skin type and state (*P* = 0.02) showed that freezing decreased the *F*_Insertion_ in porcine skin from 0.105 N (fresh) to 0.086 N (frozen), but did not affect the *F*_Insertion_ in human skin. *F*_Insertion_ decreased significantly as a function of RH for all skin samples from 0.107 N (35% RH) to 0.096 N (100% RH); but, in individual skin groups, decreases in *F*_Insertion_ with increasing RH were not significant. The overall decrease in *F*_Insertion_ for fresh human and porcine skin followed similar trends as those measured for *S* (Fig. 2c) and was correlated with the UTS of fresh human and porcine skin^3^. The influence of RH on in-plane mechanical properties of skin differed from out-of-plane properties^3^. Since microneedle insertions applied in-plane stress (tension) on the skin, and corneocytes in the viable epidermis and the SC are naturally stacked on top of each other in an out-of-plane orientation (similar to a brick-mortar structure; Fig. 1a), the layers appeared to provide lower rigidity and more flexibility in-plane. This arrangement of the corneocytes allowed them to slide on and pass each other during in-plane deformation (during microneedle insertion), which is facilitated by hydration^3^,^15^. In human samples, the *F*_Insertion_ of frozen skin remained unaffected by RH compared to the fresh state, while the *F*_Insertion_ decreased slightly in frozen pig skin by ~21% (35% RH; *P* = 0.003) and ~24% (100% RH; *P* = 0.09). This could be attributed to slightly higher moisture retention in porcine skin (35–68% by volume^37^) compared to human skin (24–67% by volume^38^) especially at physiological, low-humidity conditions, and the effect of freezing on the degradation of corneodesmosomes in the SC^36^.

The *D*_Insertion_ of a microneedle was significantly dependent on skin type, state and RH condition (*P* < 0.0001 for all treatments; Fig. 2d; Table 2). The *D*_Insertion_ were significantly higher for human skin (1343 μm) than porcine skin (912 μm). Freezing of skin yielded a higher *D*_Insertion_ (1462 μm) across all skin samples compared to the fresh state (793 μm), resulting therefore in softening of the skin (lower *S*, *F*_Insertion_, and *D*_Insertion_ compared to fresh skin). Similarly, the *D*_Insertion_ increased as a function of RH for all skin types, due to the swelling of cells in the skin structure, leading to softening of the skin. The relationship between the *S, F*_Insertion_, and *D*_Insertion_ was mostly that the skin sample with the highest *S* required the highest *F*_Insertion_, and the lowest *D*_Insertion_. As the skin sample with the highest *S* and *F*_Insertion_, fresh porcine skin yielded the lowest *D*_Insertion_ range (0.49–0.72 mm). Similarly, the order of *D*_Insertion_ from low to high followed fresh human skin (0.86–1.11 mm), frozen porcine skin (1.09–1.35 mm), and finally frozen human skin (1.53–1.97 mm).

The work (*W*_Insertion_) required to break the SC of the skin (estimated as one half of the product of *F*_Insertion_ and *D*_Insertion_) showed that *W*_Insertion_ increased due to freezing in human and porcine skin. In fresh human skin, the mean *W*_Insertion_ were 0.05 mJ (35% RH) and 0.06 mJ (100% RH); while freezing increased the *W*_Insertion_ to 0.09 mJ (35% RH) and 0.10 mJ (100% RH). Similarly, in fresh porcine skin, the *W*_Insertion_ were 0.03 mJ (35% RH) and 0.04 mJ (100% RH); while freezing increased the *W*_Insertion_ to 0.05 mJ (35% RH) and 0.06 mJ (100% RH). In all skin samples, the *W*_Insertion_ decreased with increasing RH, indicating an overall softening of the skin due to hydration facilitating the microneedle insertion process. In all conditions, human skin yielded higher *W*_Insertion_ compared to porcine skin, demonstrating differences in the mechanical properties between the two skin types.

## Conclusions

This study aimed to show, for the first time, a direct comparison of the mechanical properties of human and porcine skin before and after freezing, demonstrating the impact of freezing on mechanical changes that occur in skin, such as decreasing stiffness and increasing total energy required to break the stratum corneum. The methods used (microindentation and microneedle insertion profiling) helped to identify and to compare both bulk and localized mechanical properties of skin layers, while also providing insight into how human and porcine skins behave at different moisture levels, which is one of the key external stimuli influencing skin mechanics. The use of low and high relative humidity conditions to assess the mechanical characteristics of each skin sample provided information on potential avenues to manipulate skin mechanical properties to match those of fresh human skin. The findings suggested that for microneedle research, in the absence of fresh human skin, using fresh porcine skin at high humidity conditions might present a more suitable skin model (with more comparable mechanical properties to fresh human skin) than frozen human skin. This study provides a reference for mechanical studies involving skin that are challenged by difficulties obtaining fresh human skin samples, and aids in selecting the appropriate specimens for various mechanical tests.

## Materials and Methods

### Biological skin preparation

The study obtained and utilized fresh human skin excised from the abdomen region in four abdominoplasty patients under informed consent; and porcine skin excised from the abdomen of four female miniature Yucatan pigs weighing 20–30 kg (Sinclair Bio-resources, Columbia, MO). The use of discarded human skin samples for this study has been approved by the University of British Columbia’s Clinical Research Ethics Board and was performed according to Canada’s Tri-Council Policy Statement (TCPS-2: 2014) and the chapter about the use of Human Biological Materials. The use of animals for this study was approved by the University of British Columbia’s Animal Care Committee and all experimental protocols conformed to the Canadian Council on Animal Care guidelines. A portion of the freshly excised skin was immediately frozen at −80 ^o^C for 48 hours, and thawed for 1 hour before testing. The freezing conditions at −80 ^o^C were used to rapidly freeze the skin samples and to minimize the impact of ice-crystal formation on the specimens. Samples were thawed for 1 hour at room temperature to ensure all ice formed during freezing melted rapidly without damaging the skin composition. All skin samples were re-stretched to their original dimensions, cut, and mounted according to Ranamukhaarachchi *et al.*^3^ prior to testing, since skin contracted rapidly from its natural dimensions^3^. For example, the area of porcine abdominal skin samples obtained for this study contracted by 50–64% post-excision. The SC layers were separated from the subsequent layers by a mild heat treatment at 60 ^o^C for 180 s in a sealed plastic bag in a water bath^30^,^39^. Skin samples were incubated at 35% and 100% RH conditions for 20 minutes before testing. The 35% RH condition represented a physiologically-relevant humidity level for skin; while the 100% RH condition was reproducibly attained for skin testing and had shown influence on skin mechanical properties in previous analyses^17^,^31^.

### Micro-indentation of skin samples

Micro-indentation tests were performed exactly according to Ranamukhaarachchi *et al.*^3^, using a Q400 TMA instrument at 35% and 100% RH conditions^3^. Briefly, the “penetration” probe (cylindrical tip, diameter of 0.89 mm) was used to load into and unload from the skin samples at a rate of 1 N min^−1^ normal to the skin surface to a maximum force of 0.1 N for the SC samples. For ED and FT skin samples, loading and unloading was performed at 0.5 N min^−1^ to a maximum force of 0.05 N (maximum displacement less than 10% of the total thickness of the skin sample^40^). The load-displacement data were recorded at 10 Hz during the test. The initial linear portion of the unloading curve was used to calculate the slope, which estimated the out-of-plane stiffness of the skin layer. The stiffness was subsequently correlated to the Young’s modulus of skin, according to Ranamukhaarachchi *et al.*^3^.

### Microneedle insertions

Single hollow nickel microneedles were fabricated according to Mansoor *et al.*^26^. Microneedle insertion tests were performed in FT skin samples, according to Ranamukhaarachchi *et al.*^3^ using a Q400 TMA instrument at 35% RH and 100% RH^3^. Briefly, a single hollow microneedle (30 μm tip diameter, 450 μm height) was mounted onto the TMA micro-expansion probe (cylindrical tip with a diameter of 2.54 mm) and applied perpendicular to the skin surface at 10 N min^−1^ to a maximum force of 2 N. Force exerted on the skin by the microneedle as a function of its displacement into skin were recorded during the test at 10 Hz.

## Additional Information

**How to cite this article**: Ranamukhaarachchi, S. A. *et al.* A micromechanical comparison of human and porcine skin before and after preservation by freezing for medical device development. *Sci. Rep.* **6**, 32074; doi: 10.1038/srep32074 (2016).

## Supplementary Material

Supplementary Information

## Figures and Tables

**Table 1:** Effect of skin source, state, and relative humidity on the Young’s modulus of the stratum corneum, epidermis/dermis composite, and full-thickness skin determined by microindentation analysis of fresh and frozen human and porcine skin layers.

Treatments		Stratum Corneum		Epidermis/Dermis		Full-Thickness Skin	
		Mean	SD	Mean	SD	Mean	SD
Source	Human	117.12^a^	41.74	1.61^a^	0.74	1.88	1.23
	Porcine	81.28^b^	31.63	2.06^b^	2.18	1.74	1.28
	LSD	8.16		0.39		NS	
State	Fresh	93.36^a^	44.92	1.55^a^	1.23	1.71	1.22
	Frozen	105.05^b^	36.28	2.12^b^	1.93	1.91	1.29
	LSD	8.16		0.39		NS	
Relative Humidity	35%	98.69	45.42	1.77	1.27	1.87	1.19
	100%	99.71	36.6	1.9	1.94	1.75	1.33
	LSD	NS		NS		NS	
CV (%)		33.43		87.10		63.83	

The least significant difference (LSD, *P* = 0.005) value is provided for the treatments that significantly influenced the Young’s modulus of skin at a

95% confidence interval

(indicated by superscripted a and b next to the mean value).

The LSD is not provided if the *F*-value of treatment is not significant (NS).

**Table 2:** Effect of skin type, state, and relative humidity on the stiffness (N m^−1^), force (N), and displacement (μm) during microneedle insertion into fresh and frozen human and porcine skins.

Treatments		Stiffness (N m^−1^)		Force (N)		Displacement (μm)	
		Mean	SD	Mean	SD	Mean	SD
Source	Human	16.96^a^	0.52	0.107^a^	0.017	1343.40^a^	550.93
	Porcine	12.33^b^	8.31	0.096^b^	0.023	912.40^b^	500.24
	LSD	2.17		0.007		130.87	
State	Fresh	17.55^a^	8.24	0.107^a^	0.025	793.40^a^	355.06
	Frozen	11.74^b^	5.03	0.096^b^	0.015	1462.40^b^	542.88
	LSD	2.17		0.007		130.87	
Relative Humidity	35%	15.65	8.46	0.107^a^	0.023	969.78^a^	498.25
	100%	13.64	6.60	0.096^b^	0.019	1286.02^b^	590.82
	LSD	NS		0.007		130.87	
CV (%)		43.32		18.18		33.51	

The least significant difference (LSD, *P* = 0.005) is provided for the treatments that significantly impacted the stiffness, force and displacement values at a

95% confidence interval

(indicated by superscripted a and b next to the mean value).

The LSD is not provided if the *F*-value of treatment is not significant (NS).
