# Supplementary material for: A micromechanical comparison of human and porcine skin before and after preservation by freezing for medical device development
Source: Sci Rep. 2016 Aug 25;6:32074. doi: 10.1038/srep32074 (PMC4997349; doi:10.1038/srep32074)
Supplement: Supplementary Information [file srep32074-s1.doc]

**A micromechanical comparison of human and porcine skin before and after preservation by freezing for medical device development**

Ranamukhaarachchi S.A.^1,2^, Lehnert S.^2,3^, Ranamukhaarachchi S.L.^4^, Sprenger L.^5^, Schneider T.^2^, Mansoor I.^1^, Rai K.^6^, Häfeli U.O.^2^, Stoeber B.^1,7^ *

^1^ Department of Electrical and Computer Engineering, University of British Columbia, Vancouver, BC Canada V6T 1Z4

^2^ Faculty of Pharmaceutical Sciences, University of British Columbia, Vancouver, BC Canada V6T 1Z3

^3^ Institute for Food Technology and Bioprocess Engineering, Technische Universität Dresden, Dresden, Germany 01062

^4^ Faculty of Engineering, South Asian Institute of Technology and Medicine, Malabe, Sri Lanka 10115

^5^ Faculty of Mechanical Engineering, Technische Universität Dresden, 01062 Dresden, Germany

^6^ False Creek Healthcare Center, Vancouver, BC Canada V5Z 1C6

^7^ Department of Mechanical Engineering, University of British Columbia, Vancouver, BC Canada V6T 1Z4

*Corresponding author:

Dr. Boris Stoeber

E-mail: [boris.stoeber@ubc.ca](mailto:boris.stoeber@ubc.ca) Tel: +1 (604) 827-5907

Address: 2054-6250 Applied Science Lane

The University of British Columbia

Vancouver, B.C.  V6T 1Z4, Canada

The following tables provide an anatomical/compositional comparison between human and porcine skins; the ANOVA from microindentation and microneedle insertion profiling; and further information on statistically significant interactions of skin treatments identified during ANOVA.

Table S1 Anatomical and compositional differences between human and porcine skin.

| **Skin Layer** | **Attribute** | **Human Skin** | **Porcine Skin** |
| --- | --- | --- | --- |
| Stratum corneum | Total lipid content  (% weight) | 10 [^1^](#_ENREF_1) | 8 [^1^](#_ENREF_1) |
|  | Polar lipid content  (% weight of total lipids) | 70 [^1^](#_ENREF_1) | 64 [^1^](#_ENREF_1) |
|  | Non-polar lipid content  (% weight of total lipids) | 30 [^1^](#_ENREF_1) | 36 [^1^](#_ENREF_1) |
|  | Thickness | 20-40 µm [^2^](#_ENREF_2) | 12-31 µm |
| Viable Epidermis | Thickness | 50-120 µm [^5^](#_ENREF_5) | 30-100 µm [^6^](#_ENREF_6) |
|  | Differentiating corneocyte layers | 4 (stratum germinativum, spinosum, granulosum, lucidum) [^7^](#_ENREF_7) | 3 (stratum germinativum, spinosum, granulosum) [^7^](#_ENREF_7) |
| Dermis | Relative elastic fiber level | High [^7^](#_ENREF_7) | Low [^7^](#_ENREF_7) |
| Full-thickness | Moisture content  (% volume) | 24-67 [^8^](#_ENREF_8) | 35-68 [^2^](#_ENREF_2) |

Table S2 Sources of variation, degree of freedom, and mean squares for the stratum corneum, epidermis/dermis composite, and full-thickness skin obtained from the analysis of variance (ANOVA) of microindentation results for human and porcine skin layers.

| **Source** | **df** | **Stratum Corneum** | | | **Epidermis/Dermis** | | | **Full-Thickness Skin** | | |
| --- | --- | --- | --- | --- | --- | --- | --- | --- | --- | --- |
|  |  | **Mean Square** | **Sig^a^** | ***P* > F** | **Mean Square** | **Sig^a^** | ***P* > F** | **Mean Square** | **Sig^a^** | ***P* > F** |
| Source | 1 | 82186 | *** | <0.0001 | 13.28 | * | 0.0237 | 1.35 |  |  |
| State | 1 | 8736 | ** | 0.0052 | 20.65 | ** | 0.0049 | 2.76 |  |  |
| Relative Humidity (RH) | 1 | 66 |  |  | 1.11 |  |  | 0.98 |  |  |
| Source x State | 1 | 47597 | *** | <0.0001 | 0.89 |  |  | 57.19 | *** | <0.0001 |
| Source x RH | 1 | 12898 | *** | 0.0007 | 4.49 |  |  | 1.86 |  |  |
| State x RH | 1 | 6087 | * | 0.0194 | 8.81 |  | 0.0649 | 2.00 |  |  |
| Source x State x RH | 1 | 1819 |  |  | 0.65 |  |  | 5.01 |  | 0.0544 |
| Error | 248 | 1099 |  |  | 2.56 |  |  | 1.34 |  |  |
| CV% |  | 33.43 |  |  | 87.10 |  |  | 63.83 |  |  |

^a^ Statistical significance (Sig) of each source of variation or interaction thereof is indicated by asterisks as per probability of F becomes significant (*P<*0.05*, *P<*0.01**, *P<*0.001***).

Table S3 Statistically significant interactions between treatments (skin source, state, and relative humidity) influencing the out-of-plane Young’s modulus of skin layers determined by microindentation analysis at a 95% confidence interval (LSD = Least Significant Difference, SD = standard deviation; n=64).

| **Skin Layer** | **Source** | **State** | | | |
| --- | --- | --- | --- | --- | --- |
|  |  | **Fresh** | **SD** | **Frozen** | **SD** |
| Stratum Corneum | Human | 124.91 | 41.48 | 109.33 | 40.84 |
|  | Porcine | 61.81 | 18.01 | 100.76 | 30.78 |
|  | LSD | 11.48 | | | |
|  |  |  |  |  |  |
|  | **Source** | **Relative Humidity** | | | |
|  |  | **35%** | **SD** | **100%** | **SD** |
|  | Human | 123.71 | 44.38 | 110.53 | 38.13 |
|  | Porcine | 73.67 | 30.28 | 88.89 | 31.75 |
|  | LSD | 11.48 | | | |
|  |  |  |  |  |  |
|  | **State** | **Relative Humidity** | | | |
|  |  | **35%** | **SD** | **100%** | **SD** |
|  | Fresh | 97.72 | 48.84 | 88.99 | 40.54 |
|  | Frozen | 99.66 | 42.08 | 110.43 | 28.63 |
|  | LSD | 11.48 | | | |
|  | | | | | |
| Epidermis/Dermis | **State** | **Relative Humidity** | | | |
|  |  | **35%** | **SD** | **100%** | **SD** |
|  | Fresh | 1.67 | 1.4 | 1.43 | 1.02 |
|  | Frozen | 1.87 | 1.11 | 2.37 | 2.47 |
|  | LSD | 0.55 | | | |
|  | | | | | |
| Full-thickness | **Source** | **State** | | | |
|  |  | **Fresh** | **SD** | **Frozen** | **SD** |
|  | Human | 1.31 | 0.73 | 2.46 | 1.36 |
|  | Porcine | 2.11 | 1.46 | 1.37 | 0.94 |
|  | LSD | 0.4 | | | |

Table S4 Sources of variation, degree of freedom, and mean squares for the stiffness (N m^-1^), force at insertion (N), and displacement at insertion (µm) obtained from the analysis of variance (ANOVA) of microneedle insertion results for human and porcine skin layers.

| **Source** | **df** | **Stiffness** | | | **Force** | | | **Displacement** | | |
| --- | --- | --- | --- | --- | --- | --- | --- | --- | --- | --- |
|  |  | **Mean Square** | **Sig^a^** | ***P* > F** | **Mean Square** | **Sig^a^** | ***P* > F** | **Mean Square** | **Sig^a^** | ***P* > F** |
| Source | 1 | 686.72 | *** | <0.0001 | 0.0041 | *** | 0.0007 | 5944205 | *** | <0.0001 |
| State | 1 | 1079.73 | *** | <0.0001 | 0.0039 | *** | 0.0009 | 1432205 | *** | <0.0001 |
| Relative Humidity (RH) | 1 | 129.65 |  |  | 0.0035 | ** | 0.0017 | 3200332 | *** | <0.0001 |
| Source x State | 1 | 17.41 |  |  | 0.0019 | * | 0.0202 | 85328 |  |  |
| Source x RH | 1 | 130.29 |  |  | 0.0010 |  |  | 177909 |  |  |
| State x RH | 1 | 207.01 |  |  | 0.0070 |  |  | 198685 |  |  |
| Source x State x RH | 1 | 91.43 |  |  | 0.0070 |  |  | 126803 |  |  |
| Error | 120 | 38.42 |  |  | 0.0003 |  |  | 139799 |  |  |
| CV% |  | 42.32 |  |  | 18.18 |  |  | 33.15 |  |  |

^a^ Statistical significance (Sig) of each source of variation or interaction thereof is indicated by asterisks as per probability of F becomes significant (*P<*0.05*, *P<*0.01**, *P<*0.001***).

Table S5 Statistically significant interactions between treatments (skin source, state, and relative humidity) influencing the force of microneedle insertion into skin determined by microneedle insertion profiling at a 95% confidence interval (LSD = Least Significant Difference, SD = standard deviation; n=8).

| **Source** | **State** | | | |
| --- | --- | --- | --- | --- |
|  | Fresh | SD | Frozen | SD |
| Human | 0.109 | 0.021 | 0.105 | 0.011 |
| Porcine | 0.105 | 0.027 | 0.086 | 0.01 |
| LSD | 0.006 | | | |

**References:**

1 Gray, G. & Yardley, H. Lipid compositions of cells isolated from pig, human, and rat epidermis. *J. Lipid Res.* **16**, 434-440 (1975).

2 Gurjarpadhye, A. A. Effect of localized mechanical indentation on skin water content evaluated using OCT. *J. Biomed. Imaging* **2011**, 17 (2011).

3 Russell, L. M., Wiedersberg, S. & Delgado-Charro, M. B. The determination of stratum corneum thickness An alternative approach. *Eur. J. Pharm. Biopharm.* **69**, 861-870 (2008).

4 Berrutti, L. E., Singer, A. J. & McClain, S. A. Histopathologic effects of cutaneous tape stripping in pigs. *Acad. Emerg. Med.* **7**, 1349-1353 (2000).

5 Meyer, W., Schwarz, R. & Neurand, K. The skin of domestic mammals as a model for the human skin, with special reference to the domestic pig. (1978).

6 Morris, G. & Hopewell, J. Epidermal cell kinetics of the pig: a review. *Cell Prolif.* **23**, 271-282 (1990).

7 Vardaxis, N., Brans, T., Boon, M., Kreis, R. & Marres, L. Confocal laser scanning microscopy of porcine skin: implications for human wound healing studies. *J. Anat.* **190**, 601-611 (1997).

8 Suh, E.-J., Woo, Y.-A. & Kim, H.-J. Determination of water content in skin by using a FT near infrared spectrometer. *Arch. Pharmacal Res.* **28**, 458-462 (2005).
